# Breakdown of Epithelial Barrier Integrity and Overdrive Activation of Alveolar Epithelial Cells in the Pathogenesis of Acute Respiratory Distress Syndrome and Lung Fibrosis

**DOI:** 10.1155/2015/573210

**Published:** 2015-10-07

**Authors:** Shigehisa Yanagi, Hironobu Tsubouchi, Ayako Miura, Nobuhiro Matsumoto, Masamitsu Nakazato

**Affiliations:** Division of Neurology, Respirology, Endocrinology and Metabolism, Department of Internal Medicine, Faculty of Medicine, University of Miyazaki, Kiyotake, Miyazaki 889-1692, Japan

## Abstract

Individual alveolar epithelial cells (AECs) collaboratively form a tight barrier between atmosphere and fluid-filled tissue to enable normal gas exchange. The tight junctions of AECs provide intercellular sealing and are integral to the maintenance of the AEC barrier integrity. Disruption and failure of reconstitution of AEC barrier result in catastrophic consequences, leading to alveolar flooding and subsequent devastating fibrotic scarring. Recent evidences reveal that many of the fibrotic lung diseases involve AECs both as a frequent target of injury and as a driver of ongoing pathological processes. Aberrantly activated AECs express most of the growth factors and chemokines responsible for the proliferation, migration, and activation of fibroblasts. Current evidences suggest that AECs may acquire overdrive activation in the initial step of fibrosis by several mechanisms, including abnormal recapitulation of the developmental pathway, defects of the molecules essential for epithelial integrity, and acceleration of aging-related properties. Among these initial triggering events, epithelial Pten, a multiple phosphatase that negatively regulates the PI3K/Akt pathway and is crucial for lung development, is essential for the prevention of alveolar flooding and lung fibrosis through the regulation of AEC barrier integrity after injury. Reestablishment of AEC barrier integrity also involves the deployment of specialized stem/progenitor cells.

## 1. Introduction

Idiopathic pulmonary fibrosis (IPF) is a chronic, irreversible, lethal, and fibroproliferative disorder of unknown causes. Occurring in middle-aged and elderly adults, IPF develops into progressive respiratory failure [1]. IPF is pathologically characterized by scattered accumulation of fibroblasts adjacent to hyperplastic alveolar epithelial cells (AECs) and extracellular matrix deposition, which results in irreversible destruction of the lung architecture [1]. Acute respiratory distress syndrome (ARDS) is a critical syndrome consisting of acute respiratory failure associated with extensive pulmonary infiltrates [2]. The pathological characterization of ARDS includes injury to the alveolar capillary barrier that leads to the influx of protein-rich fluid into alveolar spaces and subsequent devastating lung fibrosis [3–5].

Although extensive studies have investigated the basic mechanisms responsible for the development of IPF and ARDS, no specific pharmacological treatment has been proven effective [1, 3, 6]. IPF was originally thought to be an inflammatory-driven fibrosis, that is, a fibrotic process caused by unresolved inflammatory processes. However, a growing body of evidence reveals that the pathological process of IPF may be driven by aberrantly activated AECs [1]. Aberrantly activated AECs express most of the growth factors and chemokines responsible for the proliferation, migration, and activation of fibroblasts [1, 7]. In regard to ARDS, disruption of the integrity and reconstitution of AECs barrier is an important determinant of the clinical outcome [8, 9]. Collectively, fragility of AEC barrier to lung injury, abnormal activation of AECs, and aberrant repair of AEC integrity play a critical role in the pathogenesis of IPF and ARDS. The clarification of the molecular mechanisms responsible for both susceptibility of AECs to injury and aberrant activation of AECs is important for moving toward a deeper understanding of the pathogenesis of these intractable diseases.

This review highlights the regulatory mechanisms of the AEC barrier integrity focused mainly on the role of intrinsic molecules of AECs. We first discuss the machinery of the maintenance, normal repair, and pathological fibrotic remodeling of AEC integrity. Second, we discuss the several distinct mechanisms of aberrant activation of AECs in lung fibrosis based on clinical studies and experimental studies that include AEC-specific, certain molecule-deleted mice. These mechanisms include recapitulation of the developmental pathway, defect of the molecules essential for epithelial integrity, and the acceleration of aging-related properties. We previously reported that prenatal, bronchioalveolar epithelium-specific* Pten* deleted mice exhibited thickened alveolar wall with increased number of *α*-smooth muscle actin (*α*-SMA) positive spindle cells in the alveolar septae after birth [10]. We also reported that postnatal, bronchioalveolar epithelium-specific* Pten* deleted mice exhibited exacerbated alveolar flooding and subsequent fibrotic scarring after lung injury [11]. These observations represent that deregulation of developmental pathway closely links to aberrant behavior of AECs and subsequent fibrotic scarring in the adult lung after injury. Hence, this review also focuses on the role of epithelial Pten/PI3K/Akt pathway in the mechanisms of maintenance and reconstitution of AEC integrity. Finally, we review latest works on the properties of the multiple stem/progenitor cells that play pivotal roles in the repair and replacement of the cells lost in response to AEC injury.

## 2. Maintenance, Repair, and Remodeling of AEC Barrier Integrity

The alveolar wall is lined by two types of AECs: type I and type II AECs. The flat type I AECs and capillary endothelial cells constitute the blood-air barrier that facilitates efficient gas exchange. The cuboidal type II AECs secrete surfactant that is believed to contribute to the lowering of alveolar surface tension. In addition to the individual functions of type I and type II AECs, the AEC lining layer provides a physical barrier as a unit (the so-called AECs barrier) by several concerted actions [2, 12, 13]. First, the AEC barrier functions as a mechanical barrier between the lumen and the underlying submucosa, to defend the tissue from bacteria, virus, allergens, and other noxious substances [14]. This protection is also ensured by the alveolar lining fluid, a very thin layer that results from infiltration of blood components into the alveolar space and is regulated by a pressure gradient [14]. Second, the AEC barrier participates actively in the immune response of the lung by expressing sensors to recognize danger or interruptions. It was shown that human primary type II AECs express mRNA and protein for toll-like receptor- (TLR-) 2 and TLR-4, which can be modulated by incubation with lipopolysaccharide (LPS) and tumor necrosis factor [15]. Another study reported that type I AECs from mouse lung express both TLR2 and CXCL5 in response to LPS stimulation and during pneumococcal pneumonia [16]. Third, the AEC barrier prevents the exudation of water and water-soluble materials from capillaries into the alveolar spaces, and it is responsible for the active removal of fluid from the alveolar spaces [2, 12, 13, 17]. The AEC barrier provides greater resistance to protein and fluid flux than the capillary endothelium. Indeed, in the large animal models of ARDS, damage to the endothelium alone, triggered by an intravenous and/or intra-alveolar administration of endotoxin, was insufficient to cause pulmonary edema [18]. In contrast, an injury to AECs triggered by an instillation of live bacteria resulted in severe alveolar edema [18].

Under normal conditions, epithelial cells (including AECs) establish close contacts with their neighbors through laterally located intercellular junction complexes (i.e., tight junctions (TJs) and adherens junctions), and they reside on the basement membrane (BM) [19, 20]. The TJs of AECs create a rigid intercellular seal, regulate the transportation of water and molecules between adjacent cells, and are integral to the maintenance of the integrity of the alveolar capillary barrier [21]. In mice, an administration of peptide fragment derived from* Clostridium perfringens *enterotoxin decreased the protein expression of claudin-4, a TJ protein expressed in type I AEC–type I AEC and type I AEC–type II AEC junctions, with increased pulmonary edema in response to ventilator-induced lung injury [22]. The BM supports the adhesion of AECs to the alveolar wall, providing a stable scaffold for AECs. The BM laminins and their integrin receptors are also implicated in epithelial cell differentiation [23]. Primary murine AECs cultured on laminin maintained an epithelial phenotype [24]. In contrast, on fibronectin, AECs rapidly lost surfactant expression and spread extensively [24]. Injury to the lung parenchyma and subsequent loss of AECs lead to the deposition of a provisional extracellular matrix, which is conducive to the ingrowth of fibroblasts into the alveolar space [25]. The current accepted concept is that if the BM is intact and the loss of AEC integrity is limited, the provisional matrix is reabsorbed and reepithelialization of the AEC barrier occurs concurrently [2, 25–28]. An extensive loss of AECs from the alveolar wall and/or a considerable degradation of the BM will lead to the proliferation and activation of fibroblasts and excessive collagen deposition, which may result in irreversible fibrotic scarring [2, 25, 29].

## 3. Aberrant Activation of AECs in Lung Fibrosis

The elucidation of genetic disorders leading to fibrotic disease and the numerous studies of mouse models of fibrotic lung disease have revealed that many of the fibrotic diseases involve the epithelium both as a frequent target of injury and disease initiation and as a driver of ongoing pathological processes [1, 17]. In the normal repair process, activated AECs provide a milieu for subsequent repair by stabilizing the fibronectin-rich provisional matrix, by responding to proliferative signals and migrating over denuded alveolar BMs, and by releasing chemotactic factors that stimulate the migration and activation of fibroblasts [1, 17, 30]. In pathologic remodeling, these processes are assumed to be “cranked up into overdrive” and to be misguided by aberrantly activated AECs [1, 17, 30]. The histological findings of IPF lungs—including hyperplastic and hypertrophic type II AECs and the existence of large, elongated AECs—support this notion of aberrantly activated AECs. Abnormally activated AECs express most of the growth factors and chemokines responsible for the migration, proliferation, and activation of fibroblasts after lung microinjuries [1, 31–35]. One of the central actors in the fibrotic response, transforming growth factor-beta 1 (TGF-*β*1), and activators of its latent form are also produced by AECs [36, 37]. The* in situ* hybridization of human lung tissue revealed the mRNA expression of TGF-*β*1 in AECs [36]. Another study showed the increased mRNA expression of TGF-*β*1 in the lung epithelial cell line A549 under the stimulation of reactive oxygen intermediates [37]. In regard to the activator of latent form TGF-*β*1 in AECs, Munger and colleagues [38] used a bleomycin-induced lung fibrosis model and reported that latent TGF-*β*1 can be activated by binding to *α*v*β*6 integrin expressed on AECs. AECs may also contribute to the formation of the fibroblast and myofibroblast foci by the expansion of the fibroblast/myofibroblast population through the proliferation of resident mesenchymal cells [39], the attraction of circulating fibrocytes [40, 41], and stimulation of the epithelial mesenchymal transition (EMT) [11, 40, 42, 43].* In vivo* experiments using cell fate reporter mice revealed that AEC-derived myofibroblasts constitute a small fraction of the total myofibroblasts in lung fibrosis [11, 40, 42–44]. It thus remains unclear whether and how much EMT-derived fibroblasts/myofibroblasts contribute directly to collagen production in lung fibrosis. Epithelial plasticity with partial EMT reprogramming might be central to the organization of wound repair (i.e., migrating over denuded alveolar BMs); however, under chronic and unsolved stress, defects in epithelial plasticity may occur and the repair response may be corrupted, instead orchestrating fibrotic scar formation [7].

The precise mechanisms by which epithelial cells acquire overdrive activation in the initial step of organ fibrosis are unclear. Several studies suggested the possible involvement of (1) recapitulation of the developmental pathway, (2) defects of the molecules essential for epithelial integrity, and (3) the acceleration of aging-related properties in the mechanisms of the aberrant activation of AECs. As discussed in more detail in the following sections, most of these alterations caused by genetic factors or epigenetic reprogramming affected AECs and provoked an exacerbated fibrotic response without inflammation or with an inflammatory response in animal models, similar to that observed in wild-type littermates.

### 3.1. Recapitulation of the Developmental Pathway of AECs in Lung Fibrosis

**Figure 1:**
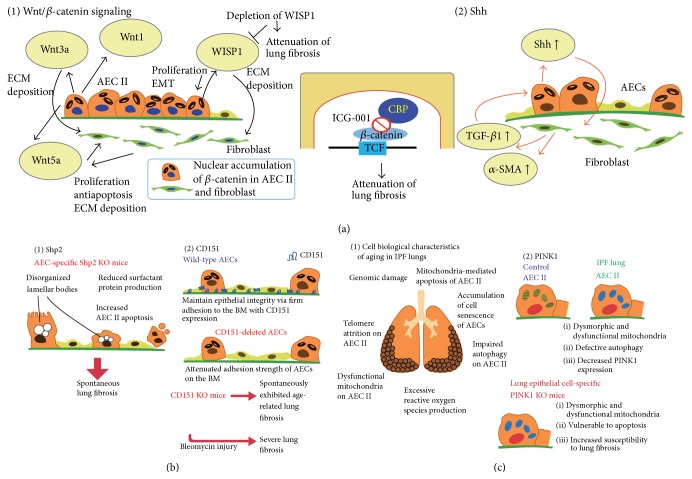
Aberrant activation of alveolar epithelial cells (AECs) in lung fibrosis. (a) Recapitulation of the developmental pathway of AECs in lung fibrosis: (1) Wnt/*β* signaling: type II AEC (AEC II) and fibroblast isolated from idiopathic pulmonary fibrosis (IPF) patients overexpress the members of the Wnt/wingless pathway and exhibit excessive nuclear accumulations of *β*-catenin. Several Wnt ligands render fibroblasts more active. WNT1-inducible signaling protein-1 (WISP1) is upregulated in AEC II in both a murine bleomycin-induced lung fibrosis model and IPF lungs. WISP1 activates AEC II and fibroblasts. Depletion of WISP1 attenuates bleomycin-induced lung fibrosis in mice. Inhibition of Wnt/*β*-catenin/CREB binding protein (CBP) also ameliorates lung fibrosis of bleomycin-treated mice. (2) Sonic hedgehog (Shh) signaling: a high expression of Shh protein is seen in the epithelial cells lining honeycomb cysts in lungs affected by IPF. TGF-*β* induced Shh expression in cultured murine AECs, whereas Shh induced TGF-*β* and alpha-smooth muscle actin (*α*-SMA) expression in cultured murine lung fibroblasts. (b) Defect of molecules essential for epithelial integrity: (1) Shp2: AEC-specific deletion of Shp2 in mice induces deregulated surfactant homeostasis, increased AEC II apoptosis, and spontaneous inflammation-independent lung fibrosis. (2) CD151: tetraspanin CD151 is expressed at the basolateral surface of AECs and is important in maintaining AEC integrity via rigid adhesion to the basement membrane (BM). CD151-deleted cultured AECs show attenuated adhesion on the BM. CD151-KO mice exhibit spontaneous age-related lung fibrosis. (c) Acceleration of aging-related properties: (1) cell biological characteristics of aging in the IPF lungs and (2) PTEN-induced putative kinase 1 (PINK1). AEC II in IPF lungs exhibits marked accumulation of dysmorphic and dysfunctional mitochondria, defective autophagy, and decreased PINK1 expression. Lung epithelial cell-specific PINK1-deleted mice show dysmorphic and dysfunctional mitochondria and vulnerability to apoptosis. PINK1 KO mice also show increased susceptibility to lung fibrosis after both MHV68 (a murine gammaherpesvirus homologous to EBV) infection and bleomycin treatment. EMT: epithelial mesenchymal transition; ECM: extracellular matrix; TCF: T-cell factor.

Wnt/*β*-catenin signaling is essential to cell fate decision, the maintenance of stem/progenitor cells, and morphogenetic processes. Results from studies of IPF lungs and a mouse model of lung fibrosis indicated that type II AECs and fibroblasts overexpress members of the Wnt/wingless pathway [45–48] (Figure 1(a)). Significant increases of Wnt3a expression were detected in type II AECs derived from IPF patients, and this increased Wnt3a expression induced fibroblast activation and collagen synthesis [49]. IPF lungs also showed extensive nuclear accumulations of *β*-catenin in AECs and fibroblasts [45]. WNT1-inducible signaling protein-1 (WISP1), which is encoded by a WNT target gene, is increased in type II AECs in both bleomycin-treated mouse lungs and human lungs affected by IPF [47]. The treatment of mouse type II AECs with recombinant WISP1 led to increased proliferation type II AECs and EMT, whereas the treatment of fibroblasts with recombinant WISP1 enhanced deposition of ECM. Interestingly, neutralizing monoclonal antibodies specific for WISP1 attenuated bleomycin-induced lung fibrosis in mice [47]. The selective inhibition of Wnt/*β*-catenin/CREB binding protein signaling ameliorated and reversed bleomycin-induced lung fibrosis in mice [50], suggesting the importance of the aberrant activation of Wnt/*β*-catenin signaling in the pathogenesis of lung fibrosis.

A linkage between the *β*-catenin signaling and cell adhesion molecules in the pathobiology of lung fibrosis was also reported. Integrin *α*3*β*1 is a laminin receptor that provides cell–BM contacts and cell–cell contacts through its interaction with the E-cadherin/*β*-catenin complex [51]. Kim and colleagues [42] reported that, after bleomycin injury, the epithelial integrin *α*3*β*1-dependent crosstalk between *β*-catenin and Smad signaling promoted myofibroblast formation and lung fibrosis. They demonstrated that mice with a lung epithelial cell-specific loss of *α*3 integrin were protected from bleomycin-induced pulmonary fibrosis. They also identified the presence of phospho-*β*-catenin/phospho-Smad2 complexes in the myofibroblasts of IPF patients, suggesting the involvement of integrin *α*3*β*1/phospho-*β*-catenin/phospho-Smad2 complexes in the pathogenesis of IPF and the importance of EMT in pathologic fibrosis.

Sonic hedgehog (Shh) is an essential morphogen for patterning during embryogenesis. It is expressed in fetal-stage epithelial cells. This developmental ligand enables cells to evade apoptosis and cell-cycle arrest, conferring a proliferative advantage. In lungs affected by IPF, a strong expression of Shh in the epithelial cells lining honeycomb cysts was reported, whereas no expressions were detected in normal lungs [52]. Hu and colleagues [53] recently provided an additional piece of evidence about the role of Shh in pulmonary fibrosis. Type II AECs of rodent bleomycin-injured lungs expressed Shh, but its expression was undetectable in lung fibroblasts. TGF-*β* induced Shh expression in cultured human AECs, whereas Shh induced TGF-*β* and *α*-SMA expression in cultured human lung fibroblasts. These results indicated the importance of epithelial mesenchymal crosstalk with a positive feedback loop mediated by epithelial-derived Shh and fibroblast/myofibroblast-derived TGF-*β* (Figure 1(a)).

### 3.2. Defect of the Molecules Essential for Epithelial Integrity

The nonreceptor protein tyrosine phosphatase Shp2 is a ubiquitously expressed intracellular enzyme and a potent inductor of branching morphogenesis and alveolarization during normal development. The AEC-specific ablation of Shp2 in mice caused a marked reduction in surfactant proteins, defective lamellar bodies, increased AEC apoptosis, and spontaneous lung fibrosis without preceding inflammation [54] (Figure 1(b)). Additionally, mice with a specific ablation of TGF-*β* receptor 2 signaling in the lung epithelium showed a decreased number of fibroblasts and protection from bleomycin-induced lung fibrosis [55, 56], despite increased inflammation [55]. These findings suggest that epithelial TGF-*β* signaling is a key player in fibrotic processes in the lung.

CD151 is a tetraspanin expressed at the basolateral surface of AECs and is important in maintaining AEC integrity via firm adhesion to the BM. Tsujino and colleagues [57] reported decreased numbers of CD151-positive AECs in patients with IPF, although most AECs in normal lungs express CD151, indicating that a downregulation of this tetraspanin may play a role in the pathogenesis of IPF.* In vitro*, CD151-knockdown type II AECs exhibited attenuated adhesion on Matrigel, enlarged morphology, and increased *α*-SMA expression. Importantly, CD151 KO mice at 30 weeks of age (i.e., “elderly” mice) demonstrated spontaneous lung fibrosis without changes in the inflammatory response. It was also demonstrated that bleomycin treatment caused exaggerated damage to AEC integrity, severe fibrosis, and high mortality in CD151 KO mice. These results indicated that individual proteins involved in AEC integrity play a critical role in the loss of epithelial integrity and the fibrotic response (Figure 1(b)).

### 3.3. Acceleration of Aging-Related Properties

IPF occurs in middle-aged and elderly adults, and its incidence increases with age; most IPF patients are older than 60 years at the time of diagnosis [1, 58–60]. However, the usual interstitial pneumonia (UIP) pattern of lung fibrosis, which is the histologic appearance of the fibrotic pattern of IPF, also occurs in a small number of young people. These adolescent interstitial lung diseases (ILDs) are characterized by a high frequency of cases due to genetic aberrations of pulmonary surfactant homeostasis. For example, gene mutations of ATP binding cassette member A3 (ABCA3), a transporter protein that is thought to be involved in the inward transport of phospholipids into lamellar bodies and to be expressed in type II AECs, are detected in young patients with the UIP pattern of ILDs [61, 62]. The authors of those reports proposed the involvement of* ABCA3* mutation in the UIP pattern of ILDs in young people and the significance of abnormal ABCA3 in the pathogenesis of ILDs in both adults and children.

Nevertheless, the trend for a predominance of IPF among middle-aged and elderly adults suggests a mechanistic link between chronological age and this disease [1, 58]. Aging is characterized by a progressive loss of functional integrity, leading to increased susceptibility to disease and death. Nine cell biological characteristics of aging are proposed recently: genomic instability, telomere erosion, epigenetic changes, loss of proteostasis, deregulated nutrient sensing, mitochondrial dysfunction, and altered intercellular communication [63]. Emerging evidence indicates that several cellular aging features are more prominent in IPF patients than in age-matched control subjects [64–68] (Figure 1(c)). Among these properties, short telomeres [65], cellular senescence [66], defective autophagy, and mitochondrial dysfunction [68] are frequently detected in type II AECs of lungs affected by IPF. The precise mechanisms by which aging-related pathways contribute to the development of IPF are under investigation. A current concept regarding the pathogenesis of IPF states that unresolved endoplasmic reticulum stress and mitochondrial dysfunction enhance the apoptotic responses of the AECs, which, due to the abnormal shortening of telomeres, have a deficient regenerative capacity. These processes, together with other aging-related changes, may be critical for the development of the disease [1, 58].

Bueno and colleagues [68] recently reported that type II AECs in the lungs of IPF patients exhibited marked accumulations of dysmorphic and dysfunctional mitochondria associated with increased expressions of an endoplasmic reticulum stress marker protein (Figure 1(c)). They also demonstrated that mice with a lung epithelial cell-specific deletion of PTEN-induced putative kinase 1 (PINK1)—a molecule that plays an important role in the maintenance of mitochondrial morphology and function—exhibited dysfunctional mitochondria in type II AECs, vulnerability to apoptosis, and the development of lung fibrosis. These findings indicated that aging and endoplasmic reticulum stress have a crucial effect on the physiology of AECs mitochondria and on susceptibility to lung fibrosis and that PINK1 in AECs has a pivotal role in the homeostasis of mitochondria and the prevention of lung fibrosis.

## 4. The Role of Epithelial Pten in the Maintenance of AEC Barrier Integrity

Pten is a multifunctional phosphatase that negatively regulates the phosphatidylinositide 3-kinase/Akt pathway and exerts a tumor-suppression action [69]. Akt is a serine/threonine-specific protein kinase that is involved in cellular survival pathways and oncogenesis. A regulatory effect of Pten on fibroblasts in lung fibrosis was reported [70, 71], and it was shown that the* Nkx-2.1*-driven Cre deletion of Pten confers resistance to airway injury [72]. The regulatory effects on epithelial cells in organ fibrosis, including lung fibrosis, remain unknown. We reported the critical roles of Pten signaling in normal mouse lung development, bronchioalveolar stem cell homeostasis, and the prevention of lung adenocarcinomas by using bronchioalveolar epithelium-specific* Pten*-deficient mice [10].

We recently identified essential roles of a Pten/Akt pathway in lung injury and repair [11]. Postnatally and bronchioalveolar epithelium-specific* Pten* deleted (*SOPten* ^Δ/Δ^) mice demonstrated severe hypoxia, high mortality, exacerbated alveolar flooding, increased alveolar permeability, and subsequent augmented lung fibrosis after lung injury. There were no significant differences in inflammatory cell accumulation or the number of apoptotic epithelial cells between *SOPten* ^Δ/Δ^ lungs and control lungs, but the *SOPten* ^Δ/Δ^ lungs demonstrated a loss of TJ morphology and a dissociation of cell-cell contacts after the injury. The levels of TJ component molecules, including claudin-4, zonula occludens-1, and occludin, were significantly attenuated in the *SOPten* ^Δ/Δ^ lungs compared to the control lungs after injury. The BM was also severely degraded in bleomycin-treated *SOPten* ^Δ/Δ^ lungs compared to the control lungs. In addition, the induction of dominant negative* PTEN* gene in lung epithelial cells led to decreased transepithelial electrical resistance (TER), a measure of TJ function, and to reduced TJ protein expression after TGF-*β*1 treatment. Taken together, these results indicated that epithelial Pten expression is vital for the prevention of acute lung injury and fibrosis by modulating the integrity of the AEC barrier.

In regard to the epithelial integrity-related molecules, we observed that the lungs of bleomycin-treated *SOPten* ^Δ/Δ^ mice showed increased expressions of phospho-Akt, phospho-S6K, Snail, matrix metalloproteinase (MMP) 2, and membrane type 1-MMP (MT1-MMP) and decreased expressions of claudin-4, E-cadherin, and laminin-*β*1. Claudin expression is not uniform throughout the alveolus; type II and type I AECs have distinct patterns of claudin expression [73, 74]. The most prominent difference is reflected by the findings that, in an analysis of purified AECs freshly isolated from rodents, type II AECs expressed over 17-fold more claudin-3 compared to type I AECs [74]. In contrast, claudin-4 expressions by type II and type I AECs were comparable at baseline [74], and the expression was upregulated during acute lung injury in the murine model of ventilator-induced lung injury [22].

Importantly, the AEC barrier integrity is regulated primarily by claudin-4 as well as TGF-*β*1 [21, 22, 75]. The overexpression of claudin-4 in primary rat AECs enhanced the barrier function [21]. The inhibition of claudin-4 expression resulted in the exacerbation of pulmonary edema in the murine model of ventilator-induced lung injury [22]. There is some circumstantial evidence that claudin-4 levels were associated with intact alveolar fluid clearance in human lungs [76]. We also detected decreased TER and a reduction of claudin-4 expression in* PTEN*-inactivated lung epithelial cells after TGF-*β*1 treatment [11]. Because Akt upregulates phospho-S6K/Snail, which in turn represses the claudin-4 expression [70], the decreased expression of claudin-4 in *SOPten* ^Δ/Δ^ lungs may be caused by the increased Akt/S6K/Snail signaling. The observation that the overexpression of Snail increased paracellular permeability by a reduction of claudin-4 expression [77] supports our findings. Since type I AECs can be generated from type II AECs [78], the claudin-4 expression might be downregulated in both type I and type II AECs of *SOPten* ^Δ/Δ^ lungs. Because phospho-Akt is upregulated in the lungs of *SOPten* ^Δ/Δ^ mice, these animals must have a great risk of acquiring severe alveolar flooding after injury. Indeed, after bleomycin-induced lung injury, Akt inactivation definitively saved *SOPten* ^Δ/Δ^ mice via the amelioration of alveolar flooding and the retention of AEC integrity [11].

## 5. The Role of Epithelial Pten in the Reconstitution of AEC Barrier Integrity

Under conditions in which epithelial Pten deficiency was already present, bleomycin challenge increased AECs' disintegrity, alveolar flooding, and subsequent lung fibrosis. To determine whether the ablation of epithelial Pten in the repair phase (after the acute injury phase) would also affect the lung fibrosis, we selectively eliminated epithelial Pten expression at the repair phase. At day 7 and day 14 (i.e., the acute phase), *SOPten* ^Δ/Δ^ (P78-84) mice, which were designed so that the Pten protein is deleted in the repair phase, showed no differences in permeability compared with control animals. These data indicated that the AEC barrier function of *SOPten* ^Δ/Δ^  (P78-84) lungs was equivalent to that of the control animals at these time points. On day 21 (i.e., the repair phase), the wild-type lungs showed a reduction of bronchoalveolar fluid protein concentrations at the baseline levels, suggesting the normal restoration of AEC barrier integrity in these mice. In contrast, *SOPten* ^Δ/Δ^  (P78-84) mice demonstrated persistent alveolar flooding with augmented lung fibrosis after injury. Because the restored integrity of AECs after their efficient repair reduces the development of fibrosis [79], *SOPten* ^Δ/Δ^ lungs are thought to fail to reepithelialize after injury.

**Figure 2:**
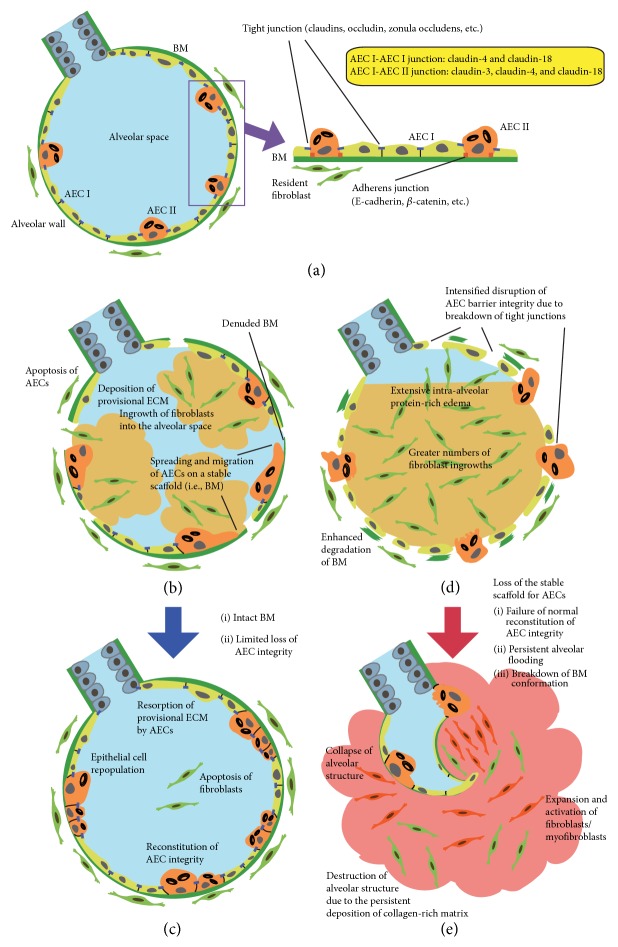
Exacerbated alveolar flooding, failure of reconstitution of alveolar epithelial cell (AEC) integrity, and subsequent augmented fibrotic scarring in epithelial cell-specific* Pten*-deficient lungs compared to the normal repair and resolution of acute lung injury in wild-type lungs. (a) Normal condition of the alveolus: type I AEC (AEC I) and type II AEC (AEC II) establish close contacts with neighbors through laterally located intercellular junctional complexes (i.e., tight junctions and adherens junctions) and reside on basement membrane (BM). Paracellular permeability depends on claudin-family tight junction proteins. Though AEC I and AEC II have distinct patterns of claudin expression, claudin-4 expression is comparable between the two types of AECs. AECs barrier integrity is primarily regulated by claudin-4. (b) Acute injury phase in wild-type lungs: injury to the lung parenchyma and loss of AECs lead to a deposition of provisional extracellular matrix (ECM), which is conducive to the ingrowth of fibroblasts. To establish the normal reconstitution of AEC integrity, AEC II spreads and migrates on a denuded BM. (c) Resolution of acute lung injury: if the BM is intact and the loss of AECs integrity is limited, the provisional ECM is reabsorbed and the reepithelialization of AEC integrity occurs. (d) Acute injury phase in epithelial cell-specific* Pten*-deficient lungs: the intensified disruption of tight junctions leads to a greater deposition of intra-alveolar provisional ECM and a subsequent induction of greater numbers of fibroblast ingrowths. Epithelial cell-specific* Pten*-deficient lungs also show considerable degradation of the BM after injury. Loss of the BM leads to a failure of the normal reconstitution of AEC integrity through deprivation of the stable scaffolding from AECs which is necessary for normal spatial orientation in cell spreading and migration. (e) Fibrotic scarring in epithelial cell-specific* Pten*-deficient lungs: failure of the reconstitution of AEC integrity and persistent alveolar flooding cause the expansion and activation of fibroblasts and myofibroblasts, which results in excessive collagen-rich matrix and fibrotic scarring.

There are two principal causes of exacerbated lung fibrosis in *SOPten* ^Δ/Δ^ lungs. First, the intensified disruption of the AEC barrier integrity due to the marked reductions of TJ expressions may lead to a greater deposition of provisional extracellular matrix in the alveoli and subsequent induction of greater number of fibroblast ingrowths (Figure 2). Second, the failure of normal reconstitution of AEC barrier integrity may also lead to the prolonged retention of protein-rich alveolar edema. The loss of AEC barrier integrity during lung injury not only leads to increased filtration of protein-rich edema into the interstitial and alveolar spaces; it also impairs the ability of the AECs to resolve the excess liquid from the alveolar space due to impaired alveolar fluid clearance [8]. In fact, the *SOPten* ^Δ/Δ^  (P78-84) mice demonstrated increased lung fibrosis with persistent alveolar flooding after injury.

**Figure 3:**
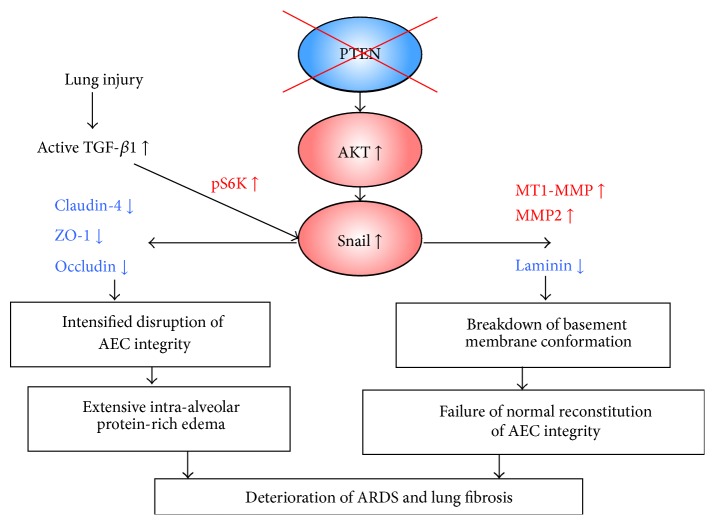
Schematic model of molecular mechanisms of deterioration of acute respiratory distress syndrome (ARDS) and lung fibrosis in epithelial cell-specific* Pten*-deficient lungs. The phosphorylation levels of Akt are elevated in the* Pten*-deficient alveolar epithelial cells (AECs) at baseline and after injury. After lung injury, epithelial cell-specific* Pten*-deficient lungs show increased expressions of pS6K, Snail, matrix metalloproteinase (MMP) 2, and membrane type 1-MMP (MT1-MMP) and decreased expressions of claudin-4, zonula occludens-1 (ZO-1), occludin, and laminin. These alterations of the expression levels of molecules in epithelial cell-specific* Pten*-deficient lungs result in intensified disruption of AEC integrity, failure of the normal reconstitution of AEC integrity, and a subsequent deterioration of ARDS and lung fibrosis. TGF-*β*1: transforming growth factor-beta 1.

After bleomycin-induced injury, *SOPten* ^Δ/Δ^ mice exhibited considerable degradation of the BM, which may be caused by the overexpressions of MT1-MMP and MMP2, which proteolyze laminin [80]. The breakdown of the BM conformation in *SOPten* ^Δ/Δ^ lungs led to a failure of the normal reconstitution of AEC integrity and to progression of lung scarring by the following potential mechanisms: collapse of the alveolar structures and fusion of adjacent BM [27] and deprivation of the stable scaffolding for AECs which is necessary for normal spatial orientation in cell spreading and migration [27, 29] (Figures 2 and 3). Reflecting the aberrant wound repair, the numbers of epithelial-derived myofibroblasts were increased in the* Pten*-knockout lungs after injury. Taken together, these results suggest that epithelial Pten in the repair phase plays an essential role in the normal reconstitution of AEC integrity and the prevention of fibrotic lung remodeling after injury.

We also detected a reduction of PTEN expression and AKT hyperactivation in the AECs of human IPF lungs. Interestingly, miR-21, a microRNA that is significantly increased in IPF, targets* PTEN* [81], suggesting that epigenetic mechanisms may be operating in the loss of epithelial integrity.

## 6. The Properties of the Epithelial Stem/Progenitor Cells and Repair of AECs Barrier

In the process of tissue regeneration, there are two ways that the replacement of the lost differentiated cells can occur in response to injury and disease: by the proliferation of common differentiated cells and/or by the deployment of specialized stem/progenitor cells [82]. Which of these pathways applies is both organ- and injury- (particular kind of damage sustained, whether it is acute or chronic, and whether it involves inflammation or immune modulation) specific [78, 82, 83]. In the lung, depending on the composition and organization of the respiratory epithelium, distinct regions of the lung contain different populations of epithelial cells that function as adult stem cells. Adult stem cells are defined by their ability to undergo long-term self-renewal and they give rise to different cell types during homeostatic turnover or cell replacement after injury [78]. In the proximal airway, the consensus from* in vivo *lineage tracing experiments is that basal cells are adult stem cells that give rise to ciliated and secretory luminal cells during postnatal growth, homeostasis, and epithelial repair [78]. In the bronchiolar region, Scfb1a1+club cells and neuroendocrine cells undergo self-renewal and generate ciliated cells (from Scfb1a1+club cells) and club cells and ciliated cells (both from neuroendocrine cells), respectively [84, 85].

In the alveolar region, the true identity of epithelial stem/progenitor cells that regenerate type I and type II AECs in the repair process is currently under debate, but recent* in vivo* genetic lineage tracing studies are gradually approaching the core of this issue. That is, distinct epithelial cell types are thought to contribute to regeneration depending on the severity of parenchymal injury [78, 82]. In the mild lung injury models including a hyperoxic lung injury model [86] and a targeted type II AEC-depletion model [87], Sftpc-positve type II AECs undergo clonal proliferation [87], generate multiple type I and/or type II AECs [86], and are long-lived [86, 87]. In contrast, in the severe lung injury models caused by H1N1 influenza viral infection [88] and bleomycin injury [23], Krt5+Trp63+ basal-like cells [88] and integrin *α*6*β*4-positive Sftpc-negative cells [23] give rise to type II AECs, not associated with the proliferation of preexisting type II AECs [23].

Vaughan and colleagues [83] recently demonstrated the regenerative role of rare lineage (CC10 and SP-C)-negative epithelial progenitor (LNEP) cells present within the normal distal lung. Quiescent LNEP cells activated a remodeling program after influenza or bleomycin injury. They proliferated and migrated widely to occupy heavily injured areas depleted of the mature lineage, at which point they differentiated towards mature epithelium, including club cells and type II AECs. That study also demonstrated the scant contribution of preexisting mature epithelial cells to such repairs, whereas LNEP cells exhibited proliferative capacity and multipotency. Notch signaling was required for the activation and maintenance of LNEP cells. The alveolar differentiation of LNEP cells was followed by a loss of Notch activity, whereas persistent Notch signaling resulted in either airway differentiation or abnormal cystic honeycombing. Importantly, epithelial cells surrounding honeycomb cysts of the lungs from patients with fibrosis also showed hyperactive Notch signaling. These data suggested that inappropriate Notch signaling may also be a major contributor to failed regeneration in fibrotic lung diseases.

## 7. Conclusion

In this review, we highlighted the machinery underlying the maintenance, repair, and pathological remodeling of AECs' integrity, the mechanisms of aberrant activation of AECs in lung fibrosis, and the properties of the multiple stem/progenitor cells that play pivotal roles in the repair and replacement of the cells lost in response to AEC injury. Many of the most common and intractable fibrotic diseases of adult lung, including ARDS and IPF, involve the epithelium both as a frequent target of injury and disease initiation and as a driver of ongoing pathobiological processes. The establishment of AEC-targeting, signaling-specific therapies and the further clarification of the spatial and temporal crosstalk between epithelial cells and mesenchymal cells may pave the way for the development of attractive therapeutic strategies against these intractable diseases.
